# Natural land cover positively correlates with COVID-19 health outcomes

**DOI:** 10.1186/s12889-023-15484-3

**Published:** 2023-03-31

**Authors:** Chao Li, Shunsuke Managi

**Affiliations:** Urban Institute & School of Engineering, Kyushu University, Fukuoka, Japan

**Keywords:** COVID-19, Prevalence, Mortality, Natural land cover, NDVI

## Abstract

**Background:**

The coronavirus disease 2019 (COVID‐19) poses special challenges for societies, as the disease causes millions of deaths. Although the direct prevention measures affect the prevalence and mortality the most, the other indirect factors, including natural environments and economics, could not be neglected. Evaluating the effect of natural land cover on COVID-19 health outcomes is an urgent and crucial public health topic.

**Methods:**

Here, we examine the relationships between natural land cover and the prevalence and mortality of COVID-19 in the United States. To probe the effects of long-term living with natural land cover, we extract county-level land cover data from 2001 to 2019. Based on statistically spatial tests, we employ the Spatial Simultaneous Autoregressive (SAC) Model to estimate natural land cover’s impact and monetary values on COVID-19 health outcomes. To examine the short-term effects of natural environments, we build a seasonal panel data set about the greenery index and COVID-19 health outcomes. The panel SAC model is used to detect the relationship between the greenery index and seasonal COVID-19 health outcomes.

**Results:**

A 1% increase in open water or deciduous forest is associated with a 0.004-death and 0.163-conformed-case, or 0.006-death and 0.099-confirmed-case decrease in every 1,000 people. Converting them into monetary value, for the mortality, a 1% increase in open water, deciduous forest, or evergreen forest in a county is equivalent to a 212-, 313-, or 219-USD increase in household income in the long term. Moreover, for the prevalence, a 1% change in open water, deciduous forest, or mixed forest is worth a 382-, 230-, or 650-USD increase in household income. Furthermore, a rational development intensity is also critical to reduce the risk of the COVID-19 pandemic. More greenery in the short term is also linked to lower prevalence and mortality.

**Conclusions:**

Our study underscores the importance of incorporating natural land cover as a means of mitigating the risks and negative consequences of future pandemics like COVID-19 and promoting overall public health.

**Supplementary Information:**

The online version contains supplementary material available at 10.1186/s12889-023-15484-3.

## Introduction

The coronavirus disease 2019 (COVID‐19) has raised serious and urgent concerns globally [1, 2]. As of Nov. 1^st^, 2021, there were almost 246.69 million confirmed cases and 5.00 million deaths due to COVID-19 worldwide (Data from WHO COVID-19 Dashboard, see https://covid19.who.int/). In the United States (U.S.), the cumulative numbers of confirmed and death cases owing to COVID-19 are 45,665,006 and 741,650, respectively, as of Nov. 1^st^, 2021 (Data from U.S. Centers for Disease Control and Prevention, CDC). Moreover, the county-level COVID-19 health outcomes, including the mortality and prevalence, vary dramatically in the United States, ranging from 0 to 10.77 deaths/1,000 capita and from 19.62 to 543.05 cases/1,000 capita. A high prevalence means higher rates of COVID-19 infection among the population, and a high mortality indicates that more people die due to COVID-19. The local population with a lower prevalence and mortality may have few other chronic diseases [3, 4] and relatively good mental health [5–7]. Therefore, from a positive perspective, COVID-19 is a cruel and dangerous filter that can identify healthy and unhealthy populations and help people to detect more factors affecting public health.

Investigations regarding the relationships between COVID-19 health outcomes and geographical factors are urgently needed to locate the high-risk areas, to slow the disease’s devastation, and to slash the risk of similar infectious disease outbreaks [8–10]. Considering the current situation, globally, eliminating COVID-19 is impossible in a short time [11]. Thus, reducing its negative impacts and risk is a critical focus. Previous studies suggest that a strong immune system is a key factor in COVID-19 patient survival [12, 13]. Chronic diseases, such as cardiovascular disease, are associated with an increased risk of death in COVID-19 patients [3]. Besides, people experience less depression and anxiety exposed to more green space during the COVID-19 pandemic [5]. Natural environments, mainly based on natural land cover, provide ecosystem services to improve physical and mental health [14–17]. Hence, the analyses on the associations of natural land cover with COVID-19 outcomes may help identify the high-death-risk areas in the COVID-19 pandemic and develop optimal land-use policies to deal with other similar public health emergencies in the future [18].

Natural environments are positively related to public health [19–21]. The severity of the COVID-19 symptoms is linked to people’s living environments [22, 23]. People living with less greenness have more medical conditions [13], like cardiovascular disease [24–26], which would ultimately exacerbate the COVID-19 symptom [3]. Numerous researchers point out that the natural land cover in the local communities is associated with health outcomes by promoting physical exercise and social connections, relieving stress, and removing air pollution, noise, and heat exposure [27–30]. The hypothesis is that increased exposure to natural land cover may strengthen the immune system and alleviate COVID-19 symptoms, related to lower prevalence and mortality. In other words, an increase in exposure to green spaces is associated with decreased risks of clinical diseases. Previous studies have partially verified this assumption. Cross-sectional studies show a positive association between proximity to parks and increased greenness with improved health outcomes [22, 23]. Furthermore, living with more greenery is linked with better mental health during the pandemic [5]. In conclusion, natural environments have the potential to enhance health and result in reduced COVID-19 prevalence and mortality.

There is a trade-off between health benefits and economic costs of boosting natural land cover, because the land is a critical resource for economic development and growth in developed areas. In this way, an evaluation of the value of natural land cover on COVID-19 is imperative, yet it has been overlooked in previous studies. To estimate the monetary valuation of environmental goods, stated preferences and revealed preference methods are widely used [31, 32]. Stated preference methods need surveys to directly ask the respondents to evaluate the monetary values of environmental goods, which is obviously inconsistent with the current topic [33]. However, the revealed preference methods are more straightforward and do not need surveys. These methods only need to investigate the relationships among variables and then utilize the estimated coefficient to calculate the marginal substitution rate between environmental goods and income based on the micro-econometric public health functions [28, 32, 34]. In other words, these methods assume that an alteration in income is critical to compensate for the change in environmental goods and vice versa.

## Materials and methods

### Materials

#### Health outcomes of COVID-19

**Fig. 1:**
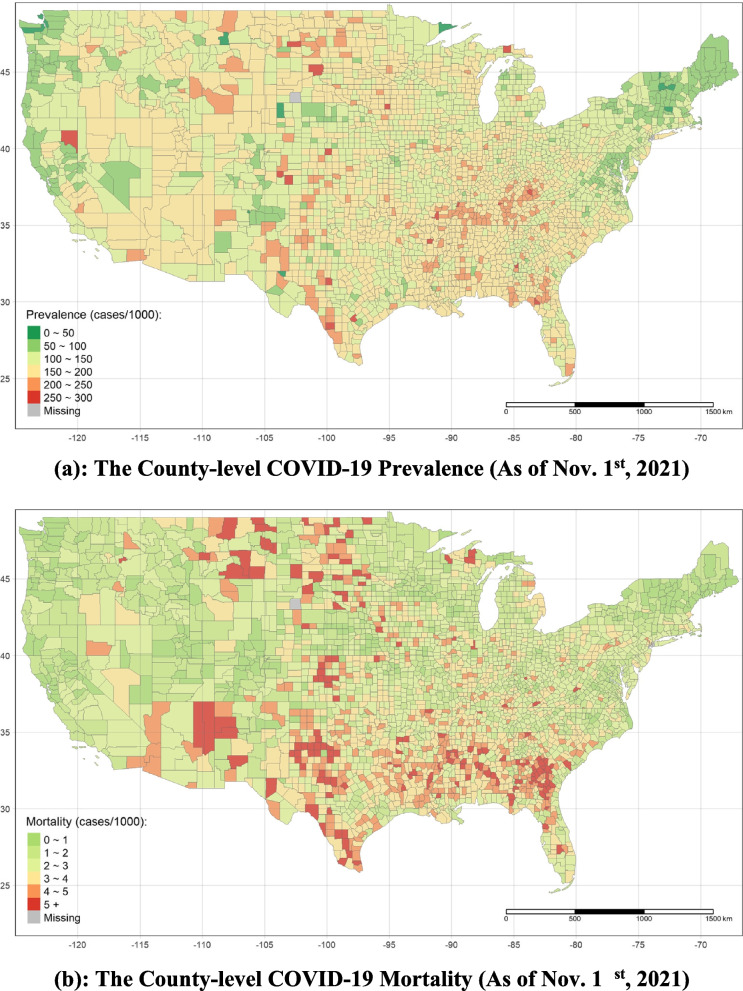
**a** The County-level COVID-19 Prevalence (As of Nov. 1^st^, 2021). **b** The County-level COVID-19 Mortality (As of Nov. 1.^st^, 2021)

Two variables, county-level prevalence and mortality, are used as the proxies for COVID-19 health outcomes. The county-level prevalence is the ratio of the confirmed cases to the total population in a certain county over a specific period, and the county-level mortality is the ratio of the deaths to the total population [35]. The unit of these two indicators is cases per 1,000 capita ($cases/\mathrm{1,000} cap$). The accumulated numbers of the confirmed cases and deaths, and population are from the CDC. To detect the impacts of long-term living with nature on COVID-19 health outcomes, the total prevalence and mortality are employed based on the accumulated numbers from the first confirmed case recorded to Nov. 1^st^, 2021. Figure 1 illustrates the spatial distribution of the total prevalence and mortality. Additionally, we calculated the quarterly prevalence and the quarterly mortality from the first quarter of 2020 to the third quarter of 2021 to examine the short-term effects of greenery. Due to some events, such as the Election, in certain months in the U.S., a tremendous monthly variation exists. To grasp the actual impacts of the environment, we, therefore, use the more stable data set, which is quarterly. It must be noted that there are reductions in the accumulated numbers of confirmed cases and deaths in several counties on some days, which might be caused by misdiagnosis, duplicate recorded by different counties, or other reasons. In the quarterly data set with more than 20,000 records, there are only no more than 50 reductions. Therefore, to avoid the negative prevalence and mortality, we force to set those reductions zero. (Table S1: Cross-Sectional Data Statistic Summary, Table S2: Panel Data Statistic Summary, Table S3: Data Source in Supplementary Materials).

### Land cover data

We extract county-level land cover data from the National Land Cover Dataset (NLCD). The NLCD archive contains eight-year data sets for the Contiguous United States (CONUS) from 2001, 2004, 2006, 2008, 2011, 2013, 2016, and 2019. These data sets include 20 land types, but there are four land types, namely, dwarf scrub, sedge, lichens, and moss, only in Alaska. In other words, in the CONUS, there are 16 other land types: open water, perennial ice, developed open space, low-intensity developed area, medium-intensity developed area, high-intensity developed area, barren land, deciduous forest, evergreen forest, mixed forest, shrub, grassland, pasture, cultivated crops, woody wetlands, and emergent herbaceous wetlands (Detailed classification description, see https://www.mrlc.gov/data/legends/national-land-cover-database-2019-nlcd2019-legend). This study considers high-intensity and medium-intensity developed areas as urban centers and urban areas, respectively. The difference among the four types of developed areas is the proportion of impervious surfaces in every grid. High-intensity developed area has over 80% impervious surface and less than 20% greenery or water. Medium-intensity developed area, low-intensity developed area, and developed open space have 50% – 80%, 20% – 50%, and less than 20% impervious surface, respectively.

The average percentages of each land type in the counties from the eight-year data set are taken as the land cover data in the analyses. At first, the total areas of each land type in the counties are obtained by tool in ArcGIS Pro 2.5.0, Tabulate Area, using the boundary shapefile from the U.S. Census Bureau. Then, they are converted into percentages, and every county in the CONUS has eight-year values. Finally, we average the values of each county. To probe the impacts of natural land cover on the COVID-19 health outcome and to get around the multicollinearity in the analyses, land cover variables, including open water, developed open space, low-intensity developed area, medium-intensity developed area, high-intensity developed area, deciduous forest, evergreen forest, mixed forest, shrub, grassland, woody wetlands, and emergent herbaceous wetlands, are put into the cross-sectional regressions. (Table S1: Cross-Sectional Data Statistic Summary).

### Normalized difference vegetation index (NDVI) data

To examine the short-term impact of the natural environment, we use the monthly NDVI data produced by the U.S. National Aeronautics and Space Administration (NASA). The NDVI is a graphical index to describe whether the observed pixel contains live green vegetation. This index range from -1 (no live green vegetation, -100%) to 1 (rife with live green vegetation, 100%). Although the panel land cover data set is desired in our study, the high-resolution raster is created every two years or longer, and there is a delay. Moreover, we also try to obtain the land cover data provided by NASA. However, NASA’s land cover products are yearly low-resolution, and 2021’s is not available. For these reasons, we eventually take the NDVI data set as the natural land cover variable in the panel regressions. We extract the monthly NDVI value based on NASA’s products, MOD13A3 (https://lpdaac.usgs.gov/products/mod13a3v006/) and MYD13A3 (https://lpdaac.usgs.gov/products/myd13a3v006/), with a 1-km resolution. Then, the quarterly average values of each county are calculated. (Table S2: Panel Data Statistic Summary).

### Other potential variables

Twenty-eight other county-level potential variables are obtained and controlled in the cross-sectional regressions. They are divided into five classes: political, demographic, socio-economic, clinical, and meteorological aspects. First, the political aspect includes five variables: the days of gatherings restrictions, the days of transport closing, the days of staying home restrictions, the days of international movement restrictions (international MoRe), and the days of internal movement restrictions (internal MoRe), from the first confirmed case recorded to Nov. 1^st^, 2021. All the political aspect confounders are acquired from the R package “COVID19” [36], based on the Oxford COVID-19 Government Response Tracker. Secondly, the demographic aspect includes six variables: the percentages of the population within specific age ranges, the percentage of black people, the percentage of Hispanic people, and the percentage of males. The U.S. Census Bureau provides the demographic data. Thirdly, the socio-economic aspect contains four variables: the unemployment rate, the median household income of counties, the poverty rate, and the percentage of the population without a high school diploma. These variables are obtained from the U.S. Department of Agriculture. Fourthly, there are eight variables in the clinical aspect: poor health rate in 2019, the average days of poor physical health in 2019, the average days of poor mental health in 2019, smoker rate in 2019, the obesity rate in 2019, physical inactivity rate in 2019, exercise opportunity rates in 2019, and the numbers of hospital beds. These data are acquired from the University of Wisconsin, School of Medicine and Public Health. Finally, the meteorological aspect contains five variables: the mean of PM_2.5_ value during 2000–2016, and means of daily temperature and relative humidity in summer (June to September) and winter (December to February) during 2000–2016. The U.S. Environmental Protection Agency provides PM_2.5_ values, and other meteorological data are downloaded and extracted from Google Climatology Lab. (Table S1: Cross-Sectional Data Statistic Summary).

Three other variables, including surface temperature, nighttime light (NTL) index, and restriction stringency score, are controlled in the panel regression to detect the relationship between the county-level NDVI and the quarterly mortality and prevalence from the first quarter in 2020 to the third quarter in 2021. The surface temperature is an average value of the monthly day-time and nighttime surface temperature extracted from other NASA’s products, MOD11C2 (https://lpdaac.usgs.gov/products/mod11c2v006/) and MOD11C2 (https://lpdaac.usgs.gov/products/myd11c2v006/) with a 0.05-arc-degree resolution. The NTL data are extracted from NASA’s products, VNP46A3 (https://ladsweb.modaps.eosdis.nasa.gov/missions-and-measurements/products/VNP46A3/). The NTL data are widely used to represent the economic status of a specific region, according to the assumption that the brighter places are generally more affluent. Because the quarterly county-level economic status and prosperity are difficult to acquire, we take the NTL index as the substitution. The restriction stringency scores of each country are obtained from the Oxford COVID-19 Government Response Tracker, which is calculated based on the restriction policies, including gathering restrictions, transport closing policies, staying home policies, internal MoRe, and international MoRe. (Table S2: Panel Data Statistic Summary, Table S3: Data Source).

## Methods

### Spatial simultaneous autoregressive (SAC) model

We utilize the SAC model to explore the connection between natural land cover and COVID-19 health outcomes in our cross-sectional analysis to estimate the long-term effect of the natural environment on the COVID-19 health outcome. The SAC model is an enhanced version of the basic model, ordinary least square (OLS), incorporating spatial consideration. We show the construction of the SAC model in a step-by-step manner to facilitate understanding. The following equation is the starting point, the OLS model, built to analyze the relationships between land cover variables and county-level health outcomes while controlling for other county-level characteristics without any additional conditions:

1 $${CHO}_{i}= {\beta }_{0}+ {{\varvec{\beta}}}_{1}{{\varvec{L}}{\varvec{C}}}_{i}+ {{\varvec{\beta}}}_{2}{{\varvec{C}}{\varvec{O}}{\varvec{N}}}_{i}+ {\varepsilon }_{i}$$

where ${CHO}_{i}$ represents the COVID-19 health outcome, either prevalence or mortality, of county $i$, ${{\varvec{L}}{\varvec{C}}}_{i}$ represents a vector of land cover data of county $i$, ${{\varvec{C}}{\varvec{O}}{\varvec{N}}}_{i}$ represents a vector of control variables of county $i$ as control variables, and ${\varepsilon }_{i}$ represents the error term. When mortality is taken as the dependent variable, ${{\varvec{C}}{\varvec{O}}{\varvec{N}}}_{i}$ would contain the prevalence of county $i$. In this model ${\beta }_{0}$, ${{\varvec{\beta}}}_{1}$ and ${{\varvec{\beta}}}_{2}$ are parameters to be estimated.

To make the model more representative of reality, we incrementally incorporate conditions into it. According to the significant Breusch-Pagan test results, heteroscedasticity exists in the abovementioned model. Because COVID-19 is an infectious disease that spreads spatially, the residuals of the OLS might also not be spatially randomly distributed. We utilize Moran’s I test for residual spatial autocorrelation. According to the test results, the significant positive values indicate spatial autocorrelation in the OLS results. In other words, the residuals of the OLS are spatially clustered. Spatial models are necessary to address the uneven distribution of residuals.

Spatial models assume the spatial correlations between a specific observation and its neighbors. The correlations might exist in the dependent variables, the independent variables, and/or the error terms [37]. If all these three correlations are significant, the OLS model, Eq. 1, will change to the Manski model, as follows:

2 $${CHO}_{i}= \rho {{\varvec{W}}}_{i}{{\varvec{N}}{\varvec{E}}{\varvec{C}}{\varvec{H}}{\varvec{O}}}_{i}+{\beta }_{0}+ {{\varvec{\beta}}}_{1}{{\varvec{L}}{\varvec{C}}}_{i}+ {{\varvec{\beta}}}_{2}{{\varvec{C}}{\varvec{O}}{\varvec{N}}}_{i}+{{\varvec{\beta}}}_{3}{{\varvec{W}}}_{i}{{\varvec{N}}{\varvec{E}}{\varvec{L}}{\varvec{C}}}_{i}+{{\varvec{\beta}}}_{4}{{\varvec{W}}}_{i}{{\varvec{N}}{\varvec{E}}{\varvec{C}}{\varvec{O}}{\varvec{N}}}_{i}+(\uplambda {{\varvec{W}}}_{i}{u}_{i}+ {\epsilon }_{i})$$

where ${{\varvec{W}}}_{i}$ is the spatial weight vector, $\rho$ is the spatial autocorrelation coefficient, ${{\varvec{N}}{\varvec{E}}{\varvec{C}}{\varvec{H}}{\varvec{O}}}_{i}$ is a vector of the COVID-19 health outcome of the neighboring counties of county $i$, ${{\varvec{N}}{\varvec{E}}{\varvec{L}}{\varvec{C}}}_{i}$ and ${{\varvec{N}}{\varvec{E}}{\varvec{C}}{\varvec{O}}{\varvec{N}}}_{i}$ are vectors of land cover variables and control variables of the neighboring counties of county $i$, $\uplambda$ is the error spatial dependence coefficient, ${u}_{i}$ is the part of error term with spatial dependence of the neighboring counties of county $i$, and ${\epsilon }_{i}$ is the part without spatial dependence. ${{\varvec{\beta}}}_{3}$ and ${{\varvec{\beta}}}_{4}$ are the parameters to be estimated, similar to ${\beta }_{0}$, ${{\varvec{\beta}}}_{1}$ and ${{\varvec{\beta}}}_{2}$. ${{\varvec{W}}}_{i}$ is built as follows:

3 $${W}_{ij}=\frac{1}{{NE}_{i}} andW_ij\;\epsilon\;W_i$$

where $${W}_{ij}$$ is the spatial weight of the neighboring county $$j$$ to county $$i$$$${NE}_{i}$$ is the number of neighboring counties of county $$i$$. Our spatial data are contiguous boundaries of each county. In this situation, if they share one boundary point, they are deemed as neighbors of each other.

The Manski model can be complicated to implement. We, therefore, perform several spatial tests to judge whether the spatial items are necessary. This test is based on the Lagrange multiplier for the spatially lagged dependence and the robust Lagrange multiplier for the error dependence. The test result shows that both spatially lagged dependence and error dependence are significant, so the items, $\rho {{\varvec{W}}}_{i}{{\varvec{N}}{\varvec{E}}{\varvec{C}}{\varvec{H}}{\varvec{O}}}_{i}$ and $\uplambda {{\varvec{W}}}_{i}{u}_{i}$, is needed in the regression. If ${{\varvec{\beta}}}_{3}{{\varvec{W}}}_{i}{{\varvec{N}}{\varvec{E}}{\varvec{L}}{\varvec{C}}}_{i}$ and ${{\varvec{\beta}}}_{3}{{\varvec{W}}}_{i}{{\varvec{N}}{\varvec{E}}{\varvec{L}}{\varvec{C}}}_{i}$ are also put into regression, the number of parameters and Akaike information criterion (AIC) will dramatically increase, eventually leading to overfitting. Thus, the SAC model is employed:

4 $${CHO}_{i}= \rho {{\varvec{W}}}_{i}{{\varvec{N}}{\varvec{E}}{\varvec{C}}{\varvec{H}}{\varvec{O}}}_{i}+{\beta }_{0}+ {{\varvec{\beta}}}_{1}{{\varvec{L}}{\varvec{C}}}_{i}+ {{\varvec{\beta}}}_{2}{{\varvec{C}}{\varvec{O}}{\varvec{N}}}_{i}+(\uplambda {{\varvec{W}}}_{i}{u}_{i}+ {\epsilon }_{i})$$

In the SAC model, the neighboring counties’ COVID-19 status affects county $i$’s, but the county $i$’s also reversely influences these neighboring counties’. Therefore, the spatial spillover function is an infinite iterative calculation. However, this function is monotonically concave and converges towards a certain value. According to the theory and previous studies, 500 iterations are enough to estimate the parameter correctly [30, 38]. In our calculation, we set the number of iterations 1000 to ensure that the convergence value is reached. Furthermore, the parameters estimated by the models involving a spatially lagged term contain two parts, direct and indirect impacts. The direct impacts are the coefficients of relationships between a given county’s independent and dependent variables, which can be directly calculated by Eq. 4 without any iteration [38]. Indirect impacts refer to the influence that an area has on its neighboring areas, which then, in turn, affects the area itself. Each time this influence travels back and forth between itself and its neighbors is an iteration. The conveyed influence decreases with each iteration, leading to convergence of the indirect impacts over time. The indirect impacts are estimated during the iterations [30, 39]. The sums of indirect impacts and direct impacts are the total impacts of the estimated parameters, regarded as the actual coefficients [39].

### Panel SAC Model

To assess the short-term impacts of the natural environments on the COVID-19 health outcome, we use the panel SAC model on the panel data set. The panel model selection process is similar to the cross-sectional model selection. The only difference is that the panel model selection process needs to choose the basic model among pooled regression model (PRM), fixed effects model (FEM), and random effects model (REM). According to the F test for individual effects, FEM is better than PRM, as the test result is significant. The significant result of the Hausman test for the panel model demonstrates that FEM is also more rational than REM. Additionally, people infected by COVID-19 are contagious. Even though having gone to hospitals, they are still able to infect other people in the relatively short term. Thus, the health outcomes are associated with the situation in the previous period. Adding a time-lagged term to the panel models is required. Accordingly, the FEM is taken as the basic model:

5 $${CHO}_{it}= {\beta }_{1}{LC}_{it}+ {{\varvec{\beta}}}_{2}{{\varvec{C}}{\varvec{O}}{\varvec{N}}}_{it}+{{{\varvec{\beta}}}_{3}{\varvec{C}}{\varvec{H}}{\varvec{O}}}_{it-1} + {a}_{i} + {\varepsilon }_{it}$$

where ${CHO}_{it}$ is the COVID-19 health outcome of county $i$ over period$t$, ${LC}_{it}$ is the NDVI indicator of county $i$ over period$t$, ${{\varvec{C}}{\varvec{O}}{\varvec{N}}}_{it}$ is a vector of control variables of county $i$ over period$t$, ${{\varvec{C}}{\varvec{H}}{\varvec{O}}}_{it-1}$ is a vector of the COVID-19 health outcome of county $i$ over period $t-1$ (if the dependent variable is the mortality, the ${{\varvec{C}}{\varvec{H}}{\varvec{O}}}_{it-1}$ should encompass both the mortality and the prevalence; otherwise, only prevalence should be included), ${a}_{i}$ is the time-invariant variable of county$i$, and ${\varepsilon }_{it}$ is an error term. ${\beta }_{1}$, ${{\varvec{\beta}}}_{2}$ and ${{\varvec{\beta}}}_{3}$ are the parameters to be estimated. Similar to OLS, FEM also assumes that the variables are spatially independent. However, the locally robust panel Lagrange multiplier tests for spatial dependence show that both spatially lagged dependence and error dependence significantly exist. Hence, the panel SAC model is applied:

6 $${CHO}_{it}= \rho {{\varvec{W}}}_{i}{{\varvec{N}}{\varvec{E}}{\varvec{C}}{\varvec{H}}{\varvec{O}}}_{it}+ {\beta }_{1}{LC}_{it}+ {{\varvec{\beta}}}_{2}{{\varvec{C}}{\varvec{O}}{\varvec{N}}}_{it}+ {{{\varvec{\beta}}}_{3}{\varvec{C}}{\varvec{H}}{\varvec{O}}}_{it-1}+ {a}_{i} + (\uplambda {{\varvec{W}}}_{i}{u}_{it}+ {\epsilon }_{it})$$

where ${{\varvec{N}}{\varvec{E}}{\varvec{C}}{\varvec{H}}{\varvec{O}}}_{it}$ is a vector of the COVID-19 health outcome of the neighboring counties of county $i$ over period $t$, ${u}_{it}$ is the part of error term with spatial dependence of the neighboring counties of county $i$ over period $t$, and ${\epsilon }_{it}$ is the part without spatial dependence.

### Monetary Value of Natural Land Cover on the COVID-19 Health Outcomes

The monetary values of natural land cover on the COVID-19 Health Outcomes are estimated to illustrate the values of the natural environment. An increase in natural land cover, such as open water and deciduous forest, is associated with a decrease in COVID-19 mortality and prevalence. Previous studies have indicated a positive relationship between greenness and COVID-19 health outcomes [22, 23]. However, there might be a trade-off between environmental benefits and economic costs. Simply boosting greenness might improve people’s health, but economic development also requires more land for construction. The monetary values can serve as a benchmark for creating land use policies, as they can provide a framework for weighing the costs and benefits of different policy options. In this study, we estimate the monetary value of increased natural land cover on COVID-19 health outcomes. Assuming that only land cover area per capita and household income change, we consider the change in household income to balance the variation in land cover area per capita as a monetary reference for land use policy formation. This method is widely used in the implicit evaluation of environmental goods, taking health evaluation as the dependent variable [17, 31, 40].

The evaluation of monetary value is based on the relationship between household income and the change in land cover, using the concept of marginal rate of substitution:

7 $$\frac{\Delta {LC}_{k}\bullet {\beta }_{1k}}{\Delta {Income}_{k}\bullet {\beta }_{Income}} = 1$$

where $\Delta {LC}_{k}$ is the change of the $k$ land cover, ${\beta }_{1k}$ represents the coefficient of the land cover $k$, $\Delta {Income}_{k}$ is the change of household income to offset the shift in land cover, and ${\beta }_{Income}$ represents the coefficient of the median household income in counties. Assuming that the change of land cover is one unit, Eq. 7 is transformed as follows:

8 $${MV}_{k} = \frac{{\beta }_{1k}}{{\beta }_{Income}} \bullet Income$$

where ${MV}_{k}$ represents the monetary value of the $k$ land cover, and $Income$ is the median household income in counties. It must be noted that the income variable in the regression is a natural logarithm, so the monetary values should be different in each county.

## Results

### Long-term Relationships between Natural Land Cover and the COVID-19 Health Outcomes

Table 1 demonstrates the result of the SAC model taking mortality as the dependent variable (Model 1). The spatially lagged dependence coefficient (*ρ*) is negative, indicating that a specific county’s mortality is negatively correlated with its neighbors’. A high mortality might threaten a region to make people carefully prevent the COVID-19. The spatially error dependence coefficient (*λ*) is positive, proposing that the ignored variables are positively associated. The pseudo R^2^ of the SAC model is 0.598, better than the OLS’s, 0.483. Natural land cover, including open water, deciduous forest, evergreen forest, is negatively related to COVID-19 mortality, whose total impacts are -0.004, -006, -0.004, respectively. The county with more natural land cover in the long term has lower mortality. Long-term living in the natural environment might improve physical health and immune system. However, the developed open space is positively associated with mortality, and its total impact is 0.020. The counties with a high proportion of developed open space usually are rural, where medical systems are relatively weak. A relatively higher proportion of natural land cover could decrease COVID-19 mortality to some degree.

**Table 1:** Result of the SAC Model Taking Mortality as the Dependent Variable (Model 1)

	Direct Impacts	Indirect Impacts	Total Impacts
Open Water (%)	-0.005646**	0.001527**	-0.004119**
	(0.002608)	(0.000749)	(0.001891)
Developed Open Space (%)	0.027805**	-0.007519**	0.020286**
	(0.013005)	(0.003622)	(0.00955)
Low Intensity Developed Area (%)	-0.011578	0.003131	-0.008447
	(0.014244)	(0.003925)	(0.010375)
Medium Intensity Developed Area (%)	-0.019557	0.005289	-0.014268
	(0.022297)	(0.006103)	(0.016285)
High Intensity Developed Area (%)	0.037524	-0.010147	0.027377
	(0.026059)	(0.007163)	(0.019078)
Deciduous Forest (%)	-0.008322***	0.00225***	-0.006071***
	(0.002366)	(0.000702)	(0.001731)
Evergreen Forest (%)	-0.005833**	0.001577**	-0.004256**
	(0.002968)	(0.000819)	(0.00218)
Mixed Forest (%)	-0.00109	0.000295	-0.000795
	(0.004576)	(0.001245)	(0.003342)
Shrub (%)	0.002565	-0.000694	0.001871
	(0.002802)	(0.000764)	(0.00205)
Grassland (%)	0.00028	-7.6e-05	0.000205
	(0.002454)	(0.000665)	(0.001795)
Woody Wetlands (%)	0.000195	-5.3e-05	0.000142
	(0.004057)	(0.001101)	(0.002966)
Emergent Herbaceous Wetlands (%)	-0.012675	0.003428	-0.009247
	(0.007617)	(0.002109)	(0.005571)
Prevalence (cap/1000)	0.013011***	-0.003519***	0.009493***
	(0.000635)	(0.000428)	(0.000589)
Gathering Restrictions (days)	-0.000167	4.5e-05	-0.000122
	(0.000348)	(9.6e-05)	(0.000254)
Transport Closing (days)	-0.000712***	0.000193***	-0.00052***
	(0.000193)	(5.8e-05)	(0.000141)
Staying Home (days)	-0.002503***	0.000677***	-0.001826***
	(0.000677)	(0.000207)	(0.000491)
Internal MoRe (days)	-0.00295	0.000798	-0.002152
	(0.006905)	(0.001871)	(0.005053)
International MoRe (days)	0.008052	-0.002178	0.005875
	(0.006843)	(0.001864)	(0.005017)
Population 15–44 (%)	-0.049464***	0.013376***	-0.036088***
	(0.010133)	(0.003108)	(0.007561)
Population 45–64 (%)	-0.004694	0.001269	-0.003425
	(0.013275)	(0.003608)	(0.009702)
Population > = 65 (%)	0.005111	-0.001382	0.003729
	(0.009735)	(0.002657)	(0.0071)
Black People (%)	-0.004495	0.001216	-0.003279
	(0.002839)	(0.000796)	(0.002063)
Hispanic People (%)	-0.016814***	0.004547***	-0.012267***
	(0.003767)	(0.001187)	(0.002739)
Male (%)	-0.036678***	0.009919***	-0.02676***
	(0.010031)	(0.002966)	(0.007377)
Unemployment Rate	0.04301**	-0.011631**	0.031379**
	(0.020824)	(0.005788)	(0.015261)
Median Household Income (logarithm)	-1.401255***	0.378932***	-1.022323***
	(0.213768)	(0.073119)	(0.159606)
Poverty Rate (%)	0.012989	-0.003512	0.009476
	(0.00787)	(0.002165)	(0.005765)
Adults Without High School Diploma (%)	0.019924***	-0.005388***	0.014536***
	(0.005513)	(0.001581)	(0.004098)
Poor Health Rate (%)	0.063922***	-0.017286***	0.046636***
	(0.017658)	(0.00532)	(0.012865)
Poor Physical Health (days)	-0.184678	0.049941	-0.134737
	(0.13012)	(0.035925)	(0.095069)
Poor Mental Health (days)	-0.230416*	0.06231*	-0.168106*
	(0.115121)	(0.032536)	(0.083802)
Adult Smoking Rate (%)	-0.039115***	0.010577***	-0.028537***
	(0.014334)	(0.00408)	(0.010518)
Obesity Rate (%)	-0.006043	0.001634	-0.004409
	(0.006535)	(0.001789)	(0.004774)
Physical Inactivity Rate (%)	0.008252	-0.002231	0.00602
	(0.006747)	(0.001842)	(0.004942)
Having Access To Exercise Opportunities (%)	-0.000961	0.00026	-0.000701
	(0.000967)	(0.000265)	(0.000706)
Hospital Beds (bed/1000)	0.004987	-0.001349	0.003638
	(0.003772)	(0.001033)	(0.002762)
Average Temperature In Summer	0.090952***	-0.024596***	0.066357***
	(0.025327)	(0.007782)	(0.018243)
Average Temperature In Winter	0.029361*	-0.00794*	0.021421**
	(0.015092)	(0.004276)	(0.010973)
Average Relative Humidity In Summer	0.019894***	-0.00538**	0.014514***
	(0.007288)	(0.002139)	(0.005277)
Average Relative Humidity In Winter	-0.010229	0.002766	-0.007463
	(0.009529)	(0.002606)	(0.006965)
PM2.5	-0.056104*	0.015172*	-0.040932*
	(0.03291)	(0.009117)	(0.024096)
Spatially lagged dependence coefficient (ρ)	-0.34506***	Log likelihood	-4093.374
Spatially error dependence coefficient (λ)	0.68623***	AIC	8276.7
Number of observations	3103	R^2^	0.5984

Note: the standard errors of the estimated parameters are list in the parentheses

^***^: *p* < 0.01; **:*p* < 0.05; *:*p* < 0.1

Additionally, several other variables listed in Table 1 are significant. Prevalence, unemployment rate, the ratio of adults without a high school diploma, poor health rate, average temperature in summer, average temperature in winter, and average relative humidity in summer are positively correlated with mortality. The positive relationship between prevalence and mortality is reasonable. More COVID-19 patients cause huge pressure on the medical systems, and this eventually leads to more deaths. The meteorological variables mentioned here depict the long-term situation rather than short-term variations. Moreover, transport closing restrictions, staying home restrictions, the proportion of population ages 15 to 44, the ratio of Hispanic people, the ratio of male, median household income, average poor mental health days, adult smoking rate, and the PM_2.5_ concentration are negatively associated with the mortality. The adult smoking rate is absolutely not the reason for the mortality reduction. We are concerned that its impacts are masked by other variables. According to the correlation test between median household income and adult smoking rate, their correlation is strongly negative (-0.661). The total impact of median household income is much greater than the adult smoking rate’s. In this way, the result is still acceptable. The situation of PM_2.5_ concentration is similar. The high PM_2.5_ concentration is harmful, which could not decrease COVID-19 mortality. There is a significantly strong correlation (0.516) between PM_2.5_ and average relative humidity in summer.

Table 2 illustrates the result of the SAC model taking prevalence as the dependent variable (Model 2). The spatially lagged dependence coefficient (*ρ*) is negative, and the spatially error dependence coefficient (*λ*) is positive. The pseudo R^2^ of the SAC model is 0.599, better than the OLS’s, 0.441. The relationships between open water, deciduous forest, and mixed forest and the prevalence are negative, whose coefficients are -0.164, -0.099, -0.278, respectively, tallying with the previous study [41]. However, another type of natural land cover, emergent herbaceous wetlands, is positively associated with the prevalence, and its total impact is 0.342. We delve into the spatial distribution of this land type. This land type is mainly distributed in Florida, Louisiana, Texas, and Minnesota, severely suffering from high COVID-19 prevalence (Figure S1 in Supplementary Materials). Additionally, the medium-intensity developed area strongly prevents the spread of COVID-19 since its total impact of the prevalence is -1.364. A rational development intensity could ensure sufficient medical resources in the region and enable residents to connect with the natural environment.

**Table 2:** Result of the SAC Model Taking Prevalence as the Dependent Variable (Model 2)

	Direct Impacts	Indirect Impacts	Total Impacts
Open Water (%)	-0.231923***	0.068225***	-0.163698***
	(0.072038)	(0.022512)	(0.050807)
Developed Open Space (%)	-0.172223	0.050663	-0.12156
	(0.360558)	(0.107107)	(0.254136)
Low Intensity Developed Area (%)	0.480323	-0.141298	0.339026
	(0.383814)	(0.113723)	(0.271906)
Medium Intensity Developed Area (%)	-1.931861***	0.5683***	-1.363562***
	(0.590928)	(0.180937)	(0.422798)
High Intensity Developed Area (%)	0.8496	-0.249929	0.599672
	(0.696589)	(0.206108)	(0.493748)
Deciduous Forest (%)	-0.139624**	0.041074**	-0.098551**
	(0.066787)	(0.0202)	(0.047203)
Evergreen Forest (%)	-0.080339	0.023634	-0.056706
	(0.084448)	(0.024909)	(0.059795)
Mixed Forest (%)	-0.394239***	0.115974***	-0.278265***
	(0.133787)	(0.041794)	(0.094123)
Shrub (%)	-0.110582	0.03253	-0.078052
	(0.087635)	(0.026256)	(0.061774)
Grassland (%)	-0.03096	0.009108	-0.021853
	(0.073891)	(0.021978)	(0.05206)
Woody Wetlands (%)	-0.025476	0.007494	-0.017982
	(0.122301)	(0.0362)	(0.086342)
Emergent Herbaceous Wetlands (%)	0.485201**	-0.142733**	0.342469**
	(0.224188)	(0.066288)	(0.160146)
Gathering Restrictions (days)	-0.000967	0.000285	-0.000683
	(0.010591)	(0.003107)	(0.007506)
Transport Closing (days)	0.017704***	-0.005208***	0.012496***
	(0.005734)	(0.001755)	(0.004088)
Staying Home (days)	-0.127064***	0.037379***	-0.089685***
	(0.019363)	(0.00683)	(0.013912)
Internal MoRe (days)	0.157108	-0.046217	0.110891
	(0.190666)	(0.056357)	(0.134936)
International MoRe (days)	0.052411	-0.015418	0.036993
	(0.188339)	(0.055714)	(0.133019)
Population 15–44 (%)	-1.532032***	0.450681***	-1.081351***
	(0.282925)	(0.092565)	(0.20586)
Population 45–64 (%)	-1.875023***	0.551579***	-1.323444***
	(0.356314)	(0.117443)	(0.257433)
Population > = 65 (%)	-2.350508***	0.691454***	-1.659054***
	(0.27225)	(0.099596)	(0.207722)
Black People (%)	-0.463024***	0.136209***	-0.326815***
	(0.080368)	(0.027638)	(0.057384)
Hispanic People (%)	0.198655*	-0.058439*	0.140216*
	(0.103038)	(0.030637)	(0.073239)
Male (%)	3.143065***	-0.924602***	2.218463***
	(0.259911)	(0.112796)	(0.206971)
Unemployment Rate	0.541061	-0.159165	0.381896
	(0.559721)	(0.165544)	(0.396054)
Median Household Income (logarithm)	-31.956257***	9.400638***	-22.555619***
	(6.330123)	(2.037901)	(4.587318)
Poverty Rate (%)	-0.422528*	0.124296*	-0.298232*
	(0.229069)	(0.068364)	(0.162612)
Adults Without High School Diploma (%)	0.128418	-0.037777	0.090641
	(0.14871)	(0.044178)	(0.105023)
Poor Health Rate (%)	0.416833	-0.122621	0.294212
	(0.506967)	(0.150881)	(0.357505)
Poor Physical Health (days)	2.526838	-0.743325	1.783513
	(3.594016)	(1.060744)	(2.542089)
Poor Mental Health (days)	-2.528876	0.743925	-1.784952
	(3.319548)	(0.981407)	(2.34709)
Adult Smoking Rate (%)	0.119984	-0.035296	0.084688
	(0.402139)	(0.118423)	(0.284535)
Obesity Rate (%)	0.363426**	-0.10691**	0.256516**
	(0.180932)	(0.054371)	(0.128235)
Physical Inactivity Rate (%)	0.559892***	-0.164705***	0.395188***
	(0.180067)	(0.055336)	(0.128256)
Having Access To Exercise Opportunities (%)	0.254658***	-0.074913***	0.179745***
	(0.025743)	(0.010531)	(0.019218)
Hospital Beds (bed/1000)	0.514691***	-0.151408***	0.363284***
	(0.103005)	(0.033162)	(0.074523)
Average Temperature In Summer	4.442997***	-1.307006***	3.135992***
	(0.774849)	(0.28612)	(0.528971)
Average Temperature In Winter	-0.593354	0.174548	-0.418806
	(0.485631)	(0.144772)	(0.343102)
Average Relative Humidity In Summer	0.510341**	-0.150128**	0.360213**
	(0.230012)	(0.069217)	(0.163197)
Average Relative Humidity In Winter	-0.350458	0.103095	-0.247363
	(0.294551)	(0.087423)	(0.208419)
PM2.5	1.657234	-0.487512	1.169722
	(1.05481)	(0.316816)	(0.744562)
Spatially lagged dependence coefficient (ρ)	-0.38423***	Log likelihood	-14,416.31
Spatially error dependence coefficient (λ)	0.76938***	AIC	28,921
Number of observations	3103	R^2^	0.5990

Note: the standard errors of the estimated parameters are list in the parentheses

^***^: *p* < 0.01; **:*p* < 0.05; *:*p* < 0.1

Several other control variables are significant and make sense in the cross-sectional analysis of the relationship between natural land cover and prevalence. Among the significant control variables, the days of transport closing, the ratio of Hispanic people, the ratio of male, obesity rate, physical inactivity rate, the ratio of people who have access to exercise opportunities, the numbers of hospital beds, average temperature in summer, and average relative humidity in summer are positively associated with the prevalence, which might favor the dispersal of the virus. However, the measures such as transport closing policies and the number of hospital beds do not increase the prevalence but rather help to control the spread of COVID-19. Since this analysis is cross-sectional, the transport closing policies are indeed affected by the COVID-19 prevalence. The counties with more hospital beds generally have more population, which are likely to cause community transmission without strict prevention policies. Obesity and physical inactivity could lead to physical health issues and reduce immunity, increasing the infection likelihood. High temperature and high humidity might be conducive to keeping the virus alive. It must be mentioned that the geographical differences cause variations in temperature and humidity here. Furthermore, staying home policies, the ratios of population ages 15 to 44, 45 to 64, and over 65, the ratio of black people, median household income, and poverty rate are negatively linked with the prevalence. Although the associations of the prevalence with the ratios of population ages 15 to 44, 45 to 64, and over 65 are all negative, the total impact of the ratio of population ages over 65 is the largest, followed by the ratio of population ages 45 to 64. Older people may pay more attention to COVID-19 prevention because they are more likely to die after the infection [13]. The richer counties also have a lower prevalence. The poverty rate is significantly correlated with the median household income (correlation coefficient: 0.846), so its real impact is masked by the median household income.

### Short-term Relationships between NDVI and the COVID-19 Health Outcomes

Table 3 shows the result of the panel SAC model taking mortality as the dependent variable (Model 3). The spatially lagged dependence coefficient is negative, while the spatial error dependence coefficient is positive. The R^2^ of the panel SAC model is 0.290, lower than the FEM’s (0.425). However, the spatially lagged dependence and error dependence tests point out that the panel SAC model is required. We, therefore, still use the panel SAC model here as the primary model. The NDVI is negatively related to mortality, whose total impact is -0.003. In other words, if the live green vegetation in the counties increases, COVID-19 mortality would decrease, and more people could survive, consistent with the previous study [42]. The prevalence in the current and previous periods causes more deaths, whose coefficients are 0.007 and 0.003, respectively. The mortality in the previous period is negatively associated with the mortality in the current period, whose coefficient is -0.084, indicating that the public could notice the caveats from the high mortality, and the governments might reallocate medical resources to prevent a further increase in deaths. The strict restriction is seemingly linked with more deaths. Of course, the higher mortality also leads to more stringent restrictions. The short-term average temperature is negatively correlated with mortality. It must be noted the short-term average temperature here is different from the temperatures used in Model 1. The variations in the temperatures used in Model 1 are mainly caused by geographical and spatial differences of the counties. For example, a county in Florida is generally warmer than a county in North Dakota. Yet, the variation of the temperatures shown here is induced by temporal differences. For instance, in a county, the average temperature during the summer season is typically higher than in the winter. Thus, the negative relationship between temperature and mortality could be explained that the COVID-19 patients are more likely to die in the winter. The NTL indicates the prosperity of counties. The NTL should be higher if the counties return to the lifestyles of pre-COVID-19 from the restrictions. If community transmission exists in those counties, it would be an outbreak, and the mortality would dramatically increase.

**Table 3:** Result of the Panel SAC Model Taking Mortality as the Dependent Variable (Model 3)

	Direct Impacts	Indirect Impacts	Total Impacts
Prevalence (cases/1000)	0.012166***	-0.00549***	0.006676***
	(0.000291)	(0.000177)	(0.00017)
Restriction Stringency	-0.000899*	0.000406*	-0.000493*
	(0.000477)	(0.000216)	(0.000261)
NDVI (%)	-0.005547***	0.002503***	-0.003044***
	(0.001112)	(0.000507)	(0.000608)
Temperature (℃)	-0.028789***	0.012991***	-0.015798***
	(0.002458)	(0.001142)	(0.001359)
NTL	0.022852***	-0.010312***	0.01254***
	(0.003825)	(0.001748)	(0.002094)
Time Lag of Prevalence (cases/1000)	0.004716***	-0.002128***	0.002588***
	(0.000319)	(0.000152)	(0.000176)
Time Lag of Mortality (cases/1000)	-0.153623***	0.069323***	-0.084301***
	(0.006957)	(0.003522)	(0.003843)
Spatially lagged dependence coefficient (ρ)	-0. 692,590***	R^2^	0.2895
Spatially error dependence coefficient (λ)	0. 8,325,688***		
Number of observations	18,612 (N:3102)		

Note: the standard errors of the estimated parameters are list in the parentheses

^***^: *p* < 0.01; **:*p* < 0.05; *:*p* < 0.1

Table 4 lists the result of the panel SAC model taking prevalence as the dependent variable (Model 4). Different from the abovementioned results, in this result, the spatially lagged dependence coefficient is positive. In the short term, the COVID-19 virus spreads spatially without too much prevention because the short-term prevalence of a specific county is strongly positively correlated with its neighbors’. Moreover, the negative spatially error dependence coefficient indicates a negative association of ignored variables. The R^2^ of the panel SAC model is 0.867, much better than the FEM’s (0.470). The negative NDVI total impact (-0.076) means the natural environment is associated with fewer patients. The high mortality leads to strict restrictions, so their relationship is positive (0.716). The low temperature reduces the infected people, whose coefficient is -2.544, aligning with the previous study [43]. The lagged prevalence is also negatively linked with the current prevalence. The high prevalence warns people to prevent the disease actively. The busy counties have a high NTL, and their residents have more chance of getting infected due to the unrigorous prevention.

**Table 4:** Result of the Panel SAC Model Taking Prevalence as the Dependent Variable (Model 4)

	Direct Impacts	Indirect Impacts	Total Impacts
Restriction Stringency	0.074928***	0.640949***	0.715876***
	(0.00503)	(0.047759)	(0.052521)
NDVI (%)	-0.023987***	-0.205187***	-0.229173***
	(0.007182)	(0.061738)	(0.068894)
Temperature (℃)	-0.266258***	-2.277629***	-2.543887***
	(0.01654)	(0.160078)	(0.175634)
NTL	0.268543***	2.297171***	2.565714***
	(0.023367)	(0.21224)	(0.234801)
Time Lag of Prevalence (cases/1000)	-0.068076***	-0.582332***	-0.650408***
	(0.004417)	(0.042491)	(0.046665)
Spatially lagged dependence coefficient (ρ)	0.8325688***	R^2^	0.8673
Spatially error dependence coefficient (λ)	-0.692590***		
Number of observations	18,612 (N:3102)		

Note: the standard errors of the estimated parameters are list in the parentheses

^***^: *p* < 0.01; **:*p* < 0.05; *:*p* < 0.1

### Impacts and Monetary Values of Natural Land Cover

With adequate natural land cover, mortality is lower. According to Model 1, a 1% increase in the ratio of open water in a county is associated with a 0.004-death decrease in the deaths due to COVID-19 per 1,000 capita in that county, shown in Table 5. Moreover, a 1% increase in the ratio of deciduous or evergreen forests is linked with a 0.006- or 0.004-death decrease in the deaths per 1,000 capita, respectively. However, a 1% increase in the ratio of developed open space is related to 0.020-death in deaths per 1,000 capita. Moreover, natural land cover and rational development intensity are also associated with fewer confirmed cases, aligning with the previous study [41]. A 1% increase in the ratio of open water, medium intensity developed area, deciduous forest, or mixed forest is correlated with a 0.164-, 1.364-, 0.099-, or 0.278-case(s) decrease in the COVID-19 confirmed cases per 1,000 capita, based on Model 2. But a 1% increase in the ratio of emergent herbaceous wetlands is associated with a 0.342-case increase. Furthermore, a 1% short-term increase in NDVI, namely greenery, leads to a 0.003-death decrease in the deaths per 1,000 capita over a certain period, according to Model 3. A 1% short-term increase in NDVI causes a 0.229-case decrease in the confirmed cases per 1,000 capita.

**Table 5:** The Impacts of Natural Land Cover Change on the Health Outcomes

Model	Land Cover Variable	Health Outcome	Impacts of Health Outcome	95% Confidence Interval
Model 1	Open Water (%)	Mortality	-0.00412	(-0.00782—-0.00041)
	Developed Open Space (%)		0.02029	(0.00157—0.039)
	Deciduous Forest (%)		-0.00607	(-0.00946—-0.00268)
	Evergreen Forest (%)		-0.00426	(-0.00853—2e-05)
Model 2	Open Water (%)	Prevalence	-0.1637	(-0.26328—-0.06412)
	Medium Intensity Developed Area (%)		-1.36356	(-2.19225—-0.53488)
	Deciduous Forest (%)		-0.09855	(-0.19107—-0.00603)
	Mixed Forest (%)		-0.27826	(-0.46275—-0.09378)
	Emergent Herbaceous Wetlands (%)		0.34247	(0.02858—0.65636)
Model 3	NDVI (%)	Mortality	-0.00304	(-0.00424—-0.00185)
Model 4	NDVI (%)	Prevalence	-0.22917	(-0.3642—-0.09414)

The value of improving natural environments is still challenging to be understood by the public without professional knowledge. To make well inform them, the coefficients of natural land cover are converted into monetary values. According to Model 1, a 1% increase in the ratio of open water, deciduous forest, or evergreen forest in a county is equivalent to an about 212-USD, 313-USD, or 219-USD increase in household income in that county, respectively, listed in Table 6. A 1% increase in the ratio of developed open space is associated with a rough 1045-USD decrease in household income. Based on Model 2, a 1% increase in the ratio of open water, medium intensity developed area, deciduous forest, or mixed forest is related to an approximately 382-USD, 3183-USD, 230-USD, or 650-USD, respectively. However, a 1% increase in emergent herbaceous wetlands correlates with a 799-USD decrease in household income.

**Table 6:** The Monetary Values of Natural Land Cover Change on the Health Outcomes

Model	Land Cover Variable	Health Outcome	Monetary Value	95% Confidence Interval
Model 1	Open Water (%)	Mortality	212	(-274—698)
	Developed Open Space (%)		-1045	(-1531—-559)
	Deciduous Forest (%)		313	(-173—799)
	Evergreen Forest (%)		219	(-267—705)
Model 2	Open Water (%)	Prevalence	382	(-104—868)
	Medium Intensity Developed Area (%)		3183	(2697—3669)
	Deciduous Forest (%)		230	(-256—716)
	Mixed Forest (%)		650	(164—1136)
	Emergent Herbaceous Wetlands (%)		-799	(-1286—-313)

## Discussion

Several natural land types are negatively associated with the spread of COVID-19 and the reduction in deaths due to COVID-19. Our results indicate that the presence of more open water and deciduous forests is linked to a lower spread of COVID-19 and fewer deaths caused by the disease in the long term. Moreover, a county with more evergreen forests and mixed forests is apt to have fewer confirmed cases or deaths. However, emergent herbaceous wetlands are seemingly positively correlated with the prevalence because of their spatial distribution. The development intensity also affects the county-level prevalence and mortality. To confirm whether these relationships happen to be statistical correlations, we further delve into the relationship between the COVID-19 health outcome and short-term average greenery, namely NDVI. The results suggest that a high NDVI in a country is associated with lower prevalence and mortality of COVID-19 in the short term, confirming the positive impact of natural environments on COVID-19 health outcomes. Furthermore, the relatively better status, such as more greenery and high household income, in a county may have a lower likelihood of experiencing COVID-19 cases and deaths, but it might also attract more people, including patients, because their direct and indirect impacts are in the opposite direction. Therefore, effective restriction policies are needed to reduce COVID-19’s impacts, even though the basic conditions in a specific place are better than the average level.

Several recent studies argue that green space may be a critical factor in the COVID-19 pandemic. An increase in urban vegetation is associated with a decrease in cumulative COVID-19 cases in the U.S. [23, 41]. The presence of parks and green spaces encourages physical activity, positively associated with human health [22, 44]. The COVID-19 infection is related to the ecological environment in South Korea [45]. However, previous studies mainly focus on a single index, such as NDVI or distance to park, and are primarily cross-sectional [22, 23]. In contrast, our study incorporates all types of land and employs a panel data set to analyze the impact of greenness on COVID-19 health outcomes, providing a more comprehensive and nuanced view that can inform land-use policies.

The importance of natural environments in the COVID-19 pandemic is illustrated in our research. According to our analyses, the natural environment may play a practical part in cutting down COVID-19 prevalence and mortality. In a way, our finding provides evidence for the previous perspectives and studies [15, 44, 46, 47]. Greenspace has effects on physical activity, obesity, mental health, cardiovascular outcomes [27], air pollution [48], and even human well-being [17, 49], especially in people living or working in high-intensity developed areas. These factors are associated with the possibility of several medical conditions, like cardiovascular and respiratory diseases, which may ultimately aggravate the severity of symptoms after being infected by the COVID-19. Based on these findings, policymakers could make the prevention and control measures more flexible to reduce the negative impacts of those strategies. An increase in natural land cover may improve public health but negatively affect the economics. To provide a clearer understanding of the trade-off between health benefits and economic cost, we estimate the monetary value of the land cover on health outcomes. To sum up, adding more green spaces to living environments should be considered in future urban planning, achieving several Sustainable Development Goals (SDGs) [50, 51].

An increase in natural land cover in living environments might not directly prevent the spread of COVID-19, but it improves public health status. In other words, with more natural land cover, people may have fewer clinical factors associated with a high risk of death infected by COVID-19 [13]. Therefore, these strategies would also prevent outbreaks of other diseases in the future. In this way, an increase in green space and reducing development intensity at least help achieve SDG 3 (good health and well-being) and 11 (sustainable cities and communities).

There are some limitations worth noting in this study. Firstly, some potential factors may be overlooked or unable to be obtained, although we have already controlled 28 county-level variables in the cross-sectional analyses. Secondly, the resolution of land cover and the lag of these data increase the uncertainty because the latest land cover data are from 2019, with a resolution of 30 m [52, 53]. Thirdly, the COVID-19 data are county-level data, possibly resulting in an ecological fallacy. Fourthly, since the models are based on FEM, all variables should be panel data with temporal variations in the panel analyses. So only a fewer variables are controlled. Future studies are better to use finer-scale or even individual-level data to detect the causal interpretation of the associations discussed in this article. The physical mechanisms that natural land cover affects the spread of contiguous diseases, similar to COVID-19, should be deeply investigated. Additionally, the specific costs and benefits of increasing natural land cover to achieve SDGs need further estimations.

## Conclusion

Our results indicate that natural land cover could reduce COVID-19 prevalence and mortality in both the long and short terms. A 1% increase in the ratio of open water, deciduous forest, or evergreen forest is linked with a 0.004-, 0.006-, or 0.004-death decrease in mortality, equivalent to a 212-, 313-, or 219-USD increase in the household income, respectively. Moreover, in terms of prevalence, a 1% increase in the ratio of open water, deciduous forest, or evergreen forest is worth 382, 230, or 650 USD, respectively. A rational development intensity of residential areas is also an effective approach to cut down deaths and confirmed cases. The relationships between short-term variations of greenery and COVID-19 health outcomes strength that natural environments could help prevent the spread of COVID-19 and reduce mortality. A 1% increase in quarterly NDVI is associated with a 0.003-death and 0.229-confirmed-case decrease per 1,000 people. Our research highlights that governments can mitigate the impacts and risks of future pandemics and improve public health by increasing the presence of natural land types such as open water and deciduous forest.

## Supplementary Information

**Additional file 1.**

## Data Availability

All data sources used in the analyses, along with fully reproducible code, are publicly available at https://github.com/MichaelChaoLi-cpu/COVID-19_and_Land_Cover_NDVI.git
