# Supplementary material for: Natural land cover positively correlates with COVID-19 health outcomes
Source: BMC Public Health. 2023 Mar 31;23:623. doi: 10.1186/s12889-023-15484-3 (PMC10064971; doi:10.1186/s12889-023-15484-3)
Supplement: Supplementary file 1 — Additional file 1. [file 12889_2023_15484_MOESM1_ESM.docx]

# Supplementary Materials

| **Table S1: Cross-sectional Data Statistic Summary** | | | | | |
| --- | --- | --- | --- | --- | --- |
| Statistic | N | Mean | St. Dev. | Min | Max |
| Mortality Rate(cases/1000) | 3,103 | 2.700 | 1.341 | 0.000 | 10.767 |
| Prevalence (cases/1000) | 3,103 | 149.992 | 36.592 | 19.622 | 543.046 |
| Open Water (%) | 3,103 | 4.795 | 11.413 | 0.000 | 90.899 |
| Developed Open Space (%) | 3,103 | 4.285 | 3.636 | 0.119 | 33.574 |
| Low Intensity Developed Area (%) | 3,103 | 2.438 | 4.067 | 0.018 | 42.512 |
| Medium Intensity Developed Area (%) | 3,103 | 1.129 | 2.822 | 0.001 | 34.421 |
| High Intensity Developed Area (%) | 3,103 | 0.465 | 1.653 | 0.00002 | 37.377 |
| Deciduous Forest (%) | 3,103 | 15.980 | 19.463 | 0.000 | 86.896 |
| Evergreen Forest (%) | 3,103 | 8.729 | 13.767 | 0.000 | 80.504 |
| Mixed Forest (%) | 3,103 | 5.254 | 7.212 | 0.000 | 56.211 |
| Shrub (%) | 3,103 | 8.431 | 18.226 | 0.000 | 97.995 |
| Grassland (%) | 3,103 | 9.385 | 17.050 | 0.001 | 97.665 |
| Woody Wetlands (%) | 3,103 | 5.458 | 9.039 | 0.000 | 67.105 |
| Emergent Herbaceous Wetlands (%) | 3,103 | 1.371 | 3.403 | 0.000 | 55.545 |
| Gathering Restrictions (days) | 3,103 | 372.568 | 118.105 | 0 | 583 |
| Transport Closing (days) | 3,103 | 207.508 | 195.640 | 0 | 578 |
| Staying Home (days) | 3,103 | 418.358 | 68.703 | 140 | 571 |
| Internal MoRe (days) | 3,103 | 559.356 | 27.756 | 332 | 584 |
| International MoRe (days) | 3,103 | 560.193 | 28.407 | 332 | 629 |
| Population 15-44 (%) | 3,103 | 35.881 | 5.053 | 15.853 | 64.848 |
| Population 45-64 (%) | 3,103 | 26.198 | 2.579 | 9.645 | 37.642 |
| Population >= 65 (%) | 3,103 | 19.812 | 4.751 | 4.859 | 58.174 |
| Black People (%) | 3,103 | 9.424 | 14.504 | 0.000 | 86.593 |
| Hispanic People (%) | 3,103 | 9.786 | 13.914 | 0.648 | 96.353 |
| Male (%) | 3,103 | 50.085 | 2.210 | 42.992 | 73.486 |
| Unemployment Rate | 3,103 | 3.960 | 1.388 | 0.700 | 18.300 |
| Median Household Income (logarithm) | 3,103 | 10.841 | 0.241 | 10.142 | 11.852 |
| Poverty Rate (%) | 3,103 | 15.165 | 6.072 | 2.600 | 48.400 |
| Adults Without High School Diploma (%) | 3,103 | 13.437 | 6.338 | 1.200 | 66.300 |
| Poor Health Rate (%) | 3,103 | 17.481 | 4.679 | 8.289 | 40.732 |
| Poor Physical Health (days) | 3,103 | 3.921 | 0.710 | 2.324 | 6.430 |
| Poor Mental Health (days) | 3,103 | 3.936 | 0.611 | 2.440 | 5.964 |
| Adult Smoking Rate (%) | 3,103 | 17.833 | 3.555 | 6.735 | 39.080 |
| Obesity Rate (%) | 3,103 | 32.085 | 4.578 | 13.600 | 49.500 |
| Physical Inactivity Rate (%) | 3,103 | 25.753 | 5.167 | 8.400 | 45.100 |
| Having Access To Exercise Opportunities (%) | 3,103 | 62.774 | 22.988 | 0.000 | 100.000 |
| Hospital Beds (bed/1000) | 3,103 | 3.037 | 4.487 | 0.000 | 99.470 |
| Average Temperature In Summer | 3,103 | 303.129 | 3.176 | 290.456 | 313.873 |
| Average Temperature In Winter | 3,103 | 280.409 | 6.602 | 264.694 | 298.340 |
| Average Relative Humidity In Summer | 3,103 | 88.977 | 9.697 | 31.643 | 99.779 |
| Average Relative Humidity In Winter | 3,103 | 87.484 | 4.789 | 58.160 | 97.673 |
| PM2.5 | 3,103 | 9.608 | 1.966 | 4.612 | 14.990 |

| **Table S2: Panel Data Statistic Summary** | | | | | | |
| --- | --- | --- | --- | --- | --- | --- |
| Period | Statistic | N | Mean | St. Dev. | Min | Max |
| 2020 Q1 | Mortality Rate(cases/1000) | 3,102 | 0.004 | 0.021 | 0 | 0 |
|  | Prevalence (cases/1000) | 3,102 | 0.150 | 0.474 | 0.000 | 10.292 |
|  | NDVI (%) | 3,102 | 38.572 | 16.125 | -0.524 | 78.896 |
|  | Temperature (℃) | 3,102 | 4.187 | 7.010 | -13.666 | 21.746 |
|  | NTL Index | 3,102 | 9.551 | 3.356 | 0.337 | 21.705 |
| 2020 Q2 | Mortality Rate(cases/1000) | 3,102 | 0.174 | 0.341 | 0.000 | 4.131 |
|  | Prevalence (cases/1000) | 3,102 | 5.030 | 7.693 | 0.000 | 132.134 |
|  | NDVI (%) | 3,102 | 57.576 | 15.466 | 13.831 | 82.701 |
|  | Temperature (℃) | 3,102 | 18.212 | 4.344 | 2.635 | 34.910 |
|  | NTL Index | 3,102 | 13.893 | 2.743 | 4.593 | 26.558 |
| 2020 Q3 | Mortality Rate(cases/1000) | 3,102 | 0.275 | 0.378 | 0.000 | 4.950 |
|  | Prevalence (cases/1000) | 3,102 | 14.653 | 10.937 | 0.000 | 142.959 |
|  | NDVI (%) | 3,102 | 68.887 | 17.221 | 12.307 | 90.035 |
|  | Temperature (℃) | 3,102 | 23.317 | 3.511 | 13.413 | 41.152 |
|  | NTL Index | 3,102 | 17.887 | 2.760 | 5.555 | 28.704 |
| 2020 Q4 | Mortality Rate(cases/1000) | 3,102 | 0.756 | 0.734 | 0 | 8 |
|  | Prevalence (cases/1000) | 3,102 | 47.908 | 22.928 | 0.000 | 259.528 |
|  | NDVI (%) | 3,102 | 48.627 | 14.988 | 12.764 | 81.384 |
|  | Temperature (℃) | 3,102 | 9.329 | 5.141 | -5.382 | 22.934 |
|  | NTL Index | 3,102 | 15.326 | 3.450 | 6.040 | 26.162 |
| 2021 Q1 | Mortality Rate(cases/1000) | 3,102 | 0.725 | 0.525 | 0.000 | 5.384 |
|  | Prevalence (cases/1000) | 3,102 | 28.651 | 11.966 | 0.000 | 154.811 |
|  | NDVI (%) | 3,102 | 37.627 | 14.443 | 6.448 | 79.555 |
|  | Temperature (℃) | 3,102 | 3.499 | 6.899 | -12.106 | 21.592 |
|  | NTL Index | 3,102 | 10.785 | 3.386 | 1.326 | 23.234 |
| 2021 Q2 | Mortality Rate(cases/1000) | 3,102 | 0.222 | 0.332 | 0.000 | 5.553 |
|  | Prevalence (cases/1000) | 3,102 | 8.802 | 6.087 | 0.000 | 96.544 |
|  | NDVI (%) | 3,102 | 57.677 | 15.363 | 11.526 | 80.733 |
|  | Temperature (℃) | 3,102 | 18.687 | 3.642 | 4.388 | 35.383 |
|  | NTL Index | 3,102 | 14.118 | 2.830 | 5.425 | 27.505 |
| 2021 Q3 | Mortality Rate(cases/1000) | 3,102 | 0.392 | 0.346 | 0.000 | 2.692 |
|  | Prevalence (cases/1000) | 3,102 | 35.594 | 17.387 | 2.160 | 113.394 |
|  | NDVI (%) | 3,102 | 69.420 | 16.966 | 11.639 | 89.066 |
|  | Temperature (℃) | 3,102 | 23.355 | 3.087 | 12.340 | 39.154 |
|  | NTL Index | 3,102 | 17.684 | 2.797 | 7.145 | 27.032 |

| **Table S3: Data Sources** | | |
| --- | --- | --- |
|  | Source | Note |
| Mortality | R Package “COVID19”  The U.S. CDC |  |
| Prevalence | R Package “COVID19”  The U.S. CDC |  |
| Boundaries of Counties | US Census Bureau https://www2.census.gov/geo/tiger/GENZ2017/shp/ | Land cover data are calculated by tool Tabulated Area in ArcGIS Pro 2.5.0 |
| Land Cover Data | Multi-Resolution Land Characteristics (MRLC) consortium https://www.mrlc.gov/ |  |
| Population | R Package “COVID19” |  |
| Gathering Restrictions | R Package “COVID19”  the Oxford COVID-19 Government Response Tracker |  |
| Transport Closing |  |  |
| Staying Home |  |  |
| Percentage Of Population 45-64 | U.S. Census Bureau https://www.census.gov/data/tables/time-series/demo/popest/2010s-counties-detail.html |  |
| Percentage Of Population ≥ 65 |  |  |
| Percentage Of Black People |  |  |
| Percentage Of Hispanic People |  |  |
| Percentage Of Males |  |  |
| Natural Logarithm Of Median Household Income In 2018 | United States Department of Agriculture https://www.ers.usda.gov/data-products/county-level-data-sets/download-data/ |  |
| Unemployment Rate In 2019 |  |  |
| Natural Logarithm Of Median House Value |  |  |
| Poverty Rate In 2018 |  |  |
| Percentage Of The Adults With Less Than High School Diploma |  |  |
| Adult Smoking Rate In 2019 | County Health Rankings & Roadmaps https://www.countyhealthrankings.org/explore-health-rankings/rankings-data-documentation |  |
| Population With Obesity Rate |  |  |
| Physical Inactivity Rate In 2019 |  |  |
| Having Access To Exercise Opportunities In 2019 |  |  |
| Mean Of Daily Temperature In Summer | Gridmet via Google Earth engine http://www.climatologylab. org/gridmet.html | 4km * 4km temperature and relative humidity predictions, summer and winter averaged during 2000-2016 |
| Mean Of Daily Temperature In Winter |  |  |
| Mean Of Relative Humidity In Summer |  |  |
| Mean Of Relative Humidity In Winter |  |  |
| PM2.5 | U.S. Environmental Protection Agency |  |
| NDVI | NASA <https://lpdaac.usgs.gov/products/mod13a3v006/> <https://lpdaac.usgs.gov/products/myd13a3v006/> | MOD13A3  MYD13A3  1-km resolution monthly raster |
| Temperature | NASA <https://lpdaac.usgs.gov/products/mod11c2v006/>  <https://lpdaac.usgs.gov/products/myd11c2v006/> | MOD11C3  MYD11C3  0.05-arc-degree resolution monthly raster |
| NTL | NASA <https://ladsweb.modaps.eosdis.nasa.gov/missions-and-measurements/products/VNP46A3/> | VNP46A3  15-arc-second resolution monthly raster |


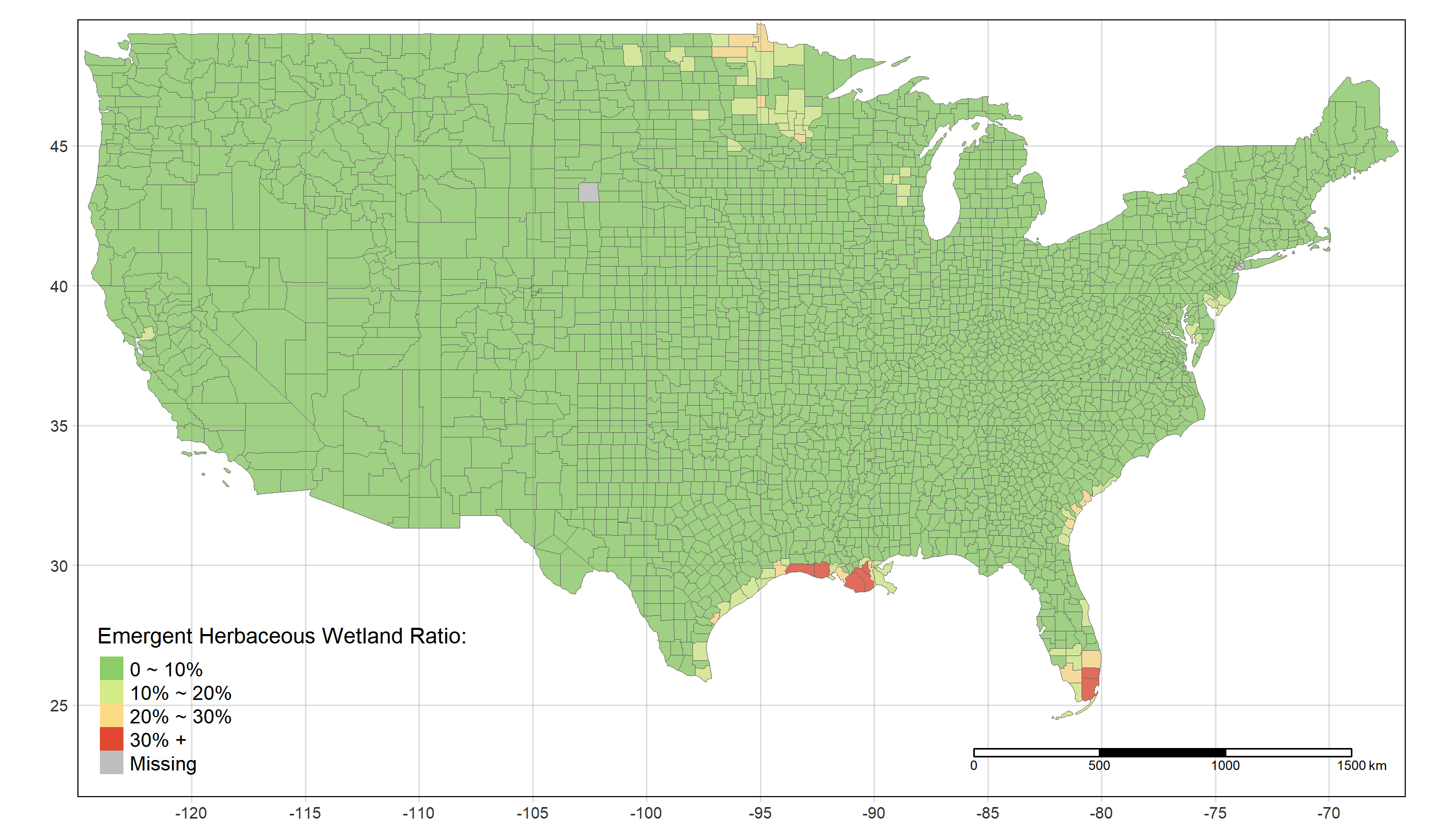


Figure S1: The Spatial Distribution of Emergent Herbaceous Wetlands Ratio
